# Non-contrast power Doppler ultrasound imaging for early assessment of trans-arterial chemoembolization of liver tumors

**DOI:** 10.1038/s41598-019-49448-8

**Published:** 2019-09-10

**Authors:** Jaime Tierney, Jennifer Baker, Anthony Borgmann, Daniel Brown, Brett Byram

**Affiliations:** 1 Vanderbilt University, Department of Biomedical Engineering, Nashville, TN 37232 USA; 2 Vanderbilt University Medical Center, Department of Radiology, Nashville, TN 37232 USA

**Keywords:** Cancer imaging, Cancer imaging, Biomedical engineering

## Abstract

Trans-arterial chemoembolization (TACE) is an important yet variably effective treatment for management of hepatic malignancies. Lack of response can be in part due to inability to assess treatment adequacy in real-time. Gold-standard contrast enhanced computed tomography and magnetic resonance imaging, although effective, suffer from treatment-induced artifacts that prevent early treatment evaluation. Non-contrast ultrasound is a potential solution but has historically been ineffective at detecting treatment response. Here, we propose non-contrast ultrasound with recent perfusion-focused advancements as a tool for immediate evaluation of TACE. We demonstrate initial feasibility in an 11-subject pilot study. Treatment-induced changes in tumor perfusion are detected best when combining adaptive demodulation (AD) and singular value decomposition (SVD) techniques. Using a 0.5 s (300-sample) ensemble size, AD + SVD resulted in a 7.42 dB median decrease in tumor power after TACE compared to only a 0.06 dB median decrease with conventional methods.

## Introduction

Liver cancer remains the 5th and 9th leading cause of cancer-related deaths globally among men and women, respectively^1^. Most affected patients (>70%) are not eligible for curative surgical treatment due to the severity of the disease upon diagnosis^2^. Trans-arterial chemoembolization (TACE) is a palliative treatment option for hepatic malignancies that do not qualify for curative transplant or resection^2–5^. TACE is also used as a down-staging or bridging tool to qualify or maintain qualification for curative treatment, respectively^2,5^. The minimally invasive procedure simultaneously delivers high doses of chemotherapy and embolizing agents to the blood supply of a tumor to provide targeted treatment and occlude the arterial supply^2–4^. TACE is variably effective, with reported response rates between 62–76%^6,7^. Low response rates can be in part due to a lack of real-time assessment of treatment adequacy. Immediate or intra-procedural treatment evaluation could identify the need for additional embolization during TACE and limit the number of repeat treatments.

Contrast-enhanced computed tomography (CE-CT) and magnetic resonance imaging (CE-MRI) are currently used to evaluate TACE treatment response^5,8^. Both modalities depict contrast agents in the blood stream as hyper-enhancement^8^. Therefore, because TACE intends to substantially reduce tumor blood perfusion, complete response is indicated by absence of enhancement in the tumor, and residual tumor manifests as hyper-enhancement in follow-up images. Both are effective evaluation tools, but follow-up imaging is not performed until at least 4–6 weeks after treatment to allow time for treatment-related imaging artifacts to subside^5,8^. For incomplete or partial response cases, this delay in treatment evaluation can result in tumor growth and a decline in the patient’s health. These treatment-related artifacts include hyper-enhancement in CE-CT and CE-MRI due to treatment-induced inflammation^9,10^. Also, one of the substances typically used for delivering chemotherapy in conventional TACE, lipiodol, mimics hyper-enhancement in CE-CT^5,9^. Contrast-enhanced ultrasound (CE-US) has been proposed as a solution for earlier treatment evaluation (during or 1–2 weeks post-treatment) because it is less affected by these artifacts in addition to being more affordable and accessible^5,10,11^. However, trapped air bubbles and drug-eluting beads (DEB) used in DEB-TACE can cause misleading hyper-enhancement in CE-US which can last up to 2 weeks post-treatment and interfere with perfusion visualization and quantification^5,11^. Additionally, all CE imaging is limited by time and dose of contrast agents in the blood stream, making any real-time treatment evaluation difficult.

Compared to CE-US, conventional non-contrast Doppler ultrasound imaging measures the signal from moving red blood cells directly as opposed to contrast agents, making it is less susceptible to misleading enhancements from the TACE treatment. For this reason, non-contrast ultrasound is a potential solution for treatment assessment during or immediately after treatment. Having immediate and reliable treatment evaluation could potentially increase treatment response rates by allowing for repeat treatments to occur during the same session, which would also be both cost and time effective for the patient and interventional radiologist. Additionally, without contrast, imaging is not constrained by time and dose of contrast agents in the blood stream, allowing for unlimited scanning time and intra-procedural imaging. Although Doppler ultrasound techniques without contrast have been shown to be limited by attenuation and tissue clutter for perfusion (i.e., the slowest blood flow in the smallest vessels) imaging applications in the past^12–14^, there have been several significant advancements to slow blood flow imaging with ultrasound in recent years that have yet to be assessed for use in TACE treatment evaluation.

Perfusion imaging with conventional Doppler techniques has been challenging mainly because of Doppler processing limitations, ineffective tissue filtering, and beamforming limitations^13,14^. Conventional color Doppler techniques are generally limited by signal-to-noise ratio (SNR) and depend on knowing the beam-to-flow angle to make accurate estimations of blood velocity^15^. However, because perfusion signal is weak and occurs in the smallest and most randomly oriented vessels, estimating perfusion velocity is particularly challenging with color Doppler techniques^16,17^. The introduction of power Doppler helped to overcome this problem because it computes the energy of the Doppler signal or the amount of moving blood as opposed to velocity, making it more sensitive to weak perfusion signal in addition to being relatively angle independent^15,18^.

Despite addressing SNR and the angle-dependency problem, tissue clutter interference with blood signal remains a barrier for perfusion applications. Tissue filtering techniques in general have been extensively studied and optimized for the purposes of conventional focused Doppler processing^19–24^. Among these techniques are adaptive tissue motion correction schemes to be implemented prior to tissue filtering in the frequency-domain^20,23^. Additionally, the most common clutter filtering methods, including infinite and finite impulse response (IIR and FIR, respectively) filters and polynomial regression filters, have been exhaustively evaluated and compared^22,24^. Similarly, eigen-based filtering techniques have been extensively studied and have been shown to be superior to more traditional techniques, albeit more computationally expensive^25–28^. However, a main limitation for all of these methods has been the trade-off between maintaining real-time frame rates and acquiring long ensembles for sufficient tissue filtering, a problem resulting from conventional Doppler beamforming which acquires a single line of a Doppler ensemble at a time^29^.

More recently, advancements in beamforming have been proposed to improve sensitivity to slow flow, including plane wave synthetic focusing and coherent flow power Doppler (CFPD)^29–31^. Plane wave applications have simultaneously overcome the trade-off between ensemble length and frame rate and have sparked renewed research in the area of slow flow imaging with ultrasound. As part of this renewal, we expanded upon the tissue motion correction schemes mentioned previously^20,23^ and developed a time-domain adaptive tissue clutter demodulation scheme on long ensemble plane wave data, accounting for both phase and amplitude modulation to suppress the bandwidth of tissue clutter prior to filtering^32^. Plane wave techniques have also led to advanced eigen-based filtering techniques that can achieve full spatio-temporal 2D and higher order singular value decompositions (SVD)^33–36^. Using mainly proof-of-concept experiments, we previously showed that combining our adaptive demodulation technique with some of the other latest improvements in tissue filtering and beamforming could potentially make imaging perfusion without contrast possible^37^.

Although non-contrast power Doppler imaging has been considered for TACE treatment evaluation in the past^12^, it has not been considered with the latest perfusion-focused improvements. Here, we propose non-contrast power Doppler imaging with adaptive demodulation in combination with improvements in beamforming and tissue filtering as a potential tool to evaluate TACE within minutes of treatment administration. For this initial study, we do not intend to demonstrate real-time capabilities but rather initial development and feasibility of these techniques for this application. Therefore, although we acquire data immediately before and after treatment, all processing is performed offline. Apart from the clinical benefits of using non-contrast ultrasound, because TACE acutely changes tumor perfusion, it also provides a unique clinical scenario to evaluate adaptive demodulation and other slow flow ultrasound algorithms. Additionally, this study tests these methods at deeper depths and with a lower transmit frequency than used in any other proof-of-concept studies. We demonstrate initial feasibility in a small pilot study.

## Results

**Figure 1:**
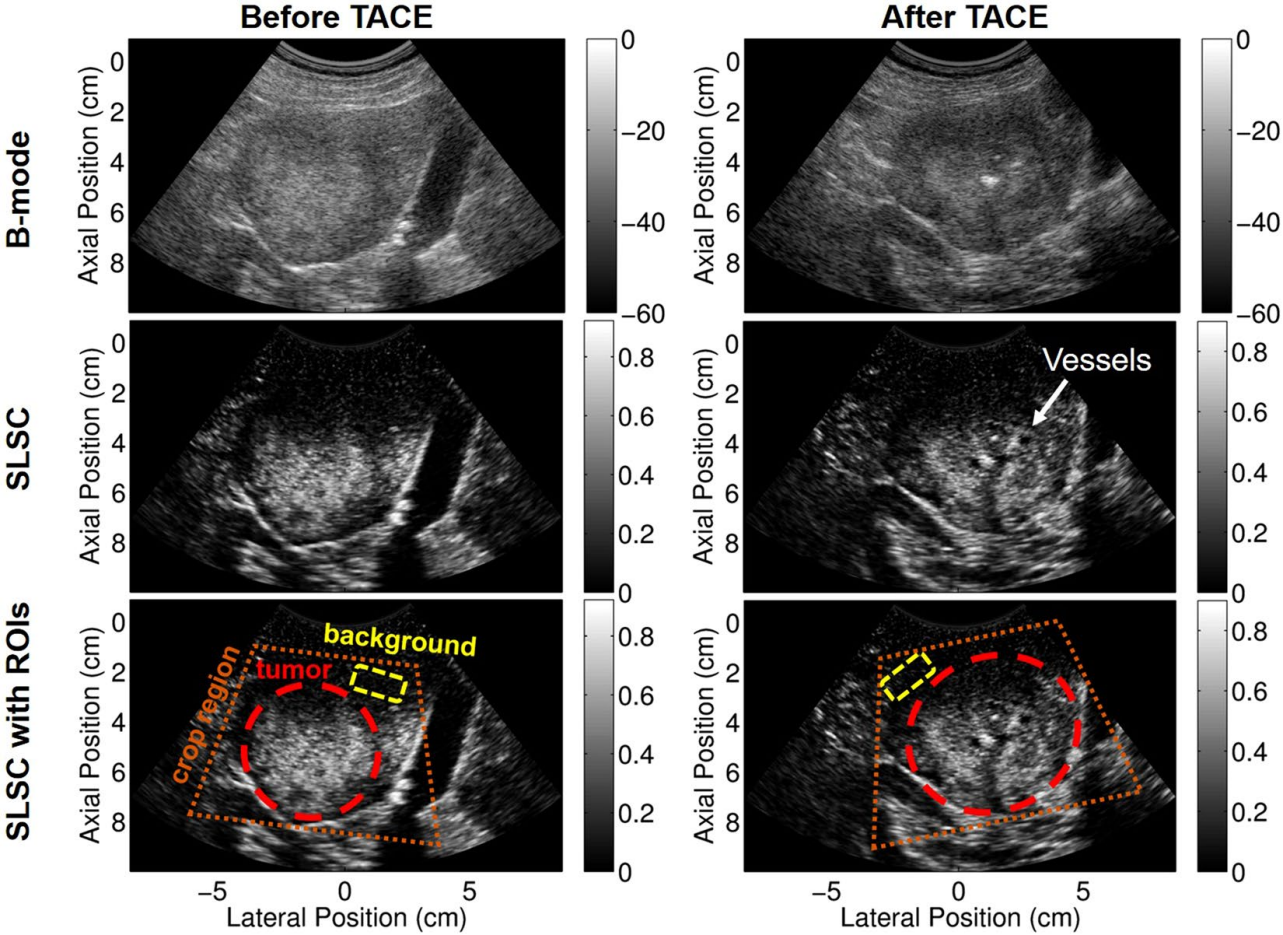
Example full field of view focused B-mode (top) and SLSC (middle and bottom) images before (left) and after (right) TACE. Potential vessels are indicated in the after TACE SLSC image that are not apparent in the B-mode. Example crop region (orange), tumor (red), and background (yellow) ROIs are displayed on focused full field of view SLSC images in the bottom row. B-mode images are scaled to individual maximums and are displayed on a dB scale. SLSC images are scaled from 0 to the maximum coherence in the image.

Focused B-mode and short-lag spatial coherence (SLSC) images were both used for identifying tumor boundaries and determining crop regions. Figure 1 shows example focused B-mode and SLSC images of subject 10 before and after TACE. The tumor is clearly seen in both the B-mode and SLSC images, but some boundaries and structures are enhanced with SLSC. Example tumor and background regions of interest (ROIs) are shown overlaid on the SLSC images for reference. Using the anatomical focused B-mode and SLSC images, the beamformed data were cropped to around the tumor for tissue filtering and power Doppler estimation, as depicted by the orange crop region in Fig. 1. This smaller field of view was used to better facilitate our global SVD filtering approach which assumes similar noise statistics throughout the input region^36^.

**Figure 2:**
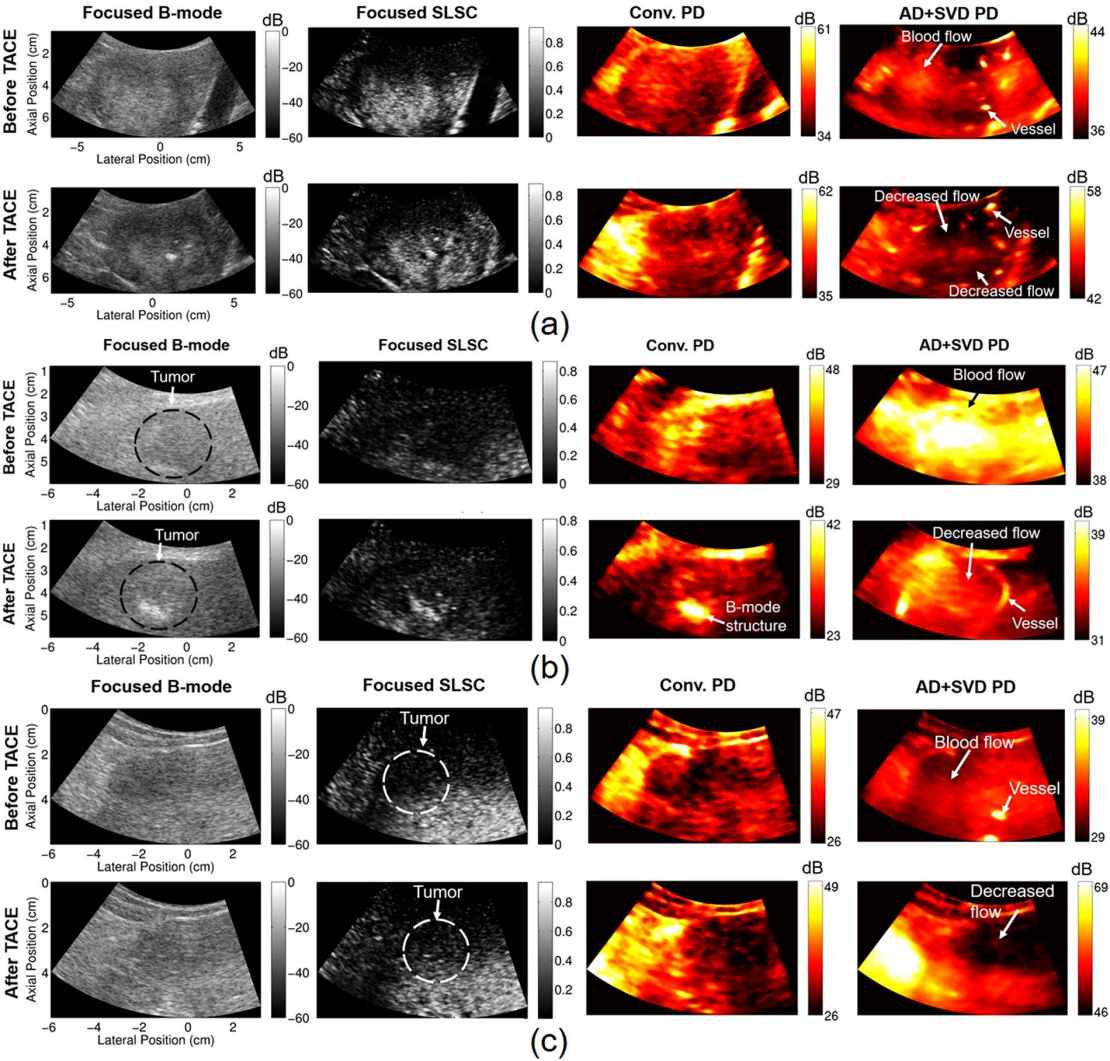
Focused B-mode, focused SLSC, and power Doppler images before and after TACE for subjects 10 (**a**), 9 (**b**), and 5 (**c**). Power Doppler (PD) images were made with conventional (Conv.) methods and with adaptive demodulation (AD) and SVD filtering. 27 ms (16 samples) and 0.5 s (300 samples) ensemble sizes were used for the conventional and advanced methods, respectively. SLSC images are scaled from 0 to the maximum coherence in the image. Potential vessels, tumor flow, and decreased flow are indicated in the AD + SVD images that are not apparent in the conventional PD images.

Qualitatively, adaptive demodulation with SVD filtering produced power Doppler images that have increased power (i.e., blood flow or perfusion) in the tumor before TACE and decreased power after (relative to surrounding tissue), which is consistent with the hypothesis for complete or partial response and is less apparent with conventional power Doppler. This is apparent in Fig. 2a–c, which show example cropped B-mode, SLSC, and power Doppler images for subjects 10, 9, and 5, respectively, before and after TACE. Power Doppler images were made with conventional methods (16-sample ensemble with IIR filtering) as well as with adaptive demodulation and SVD filtering (300-sample ensemble). Specifically, the overall power distribution within the tumor relative to the surrounding tissue in the conventional power Doppler images is similar between time points for each case. Furthermore, the structures seen in the conventional power Doppler images are highly correlated to the structure seen in the focused B-mode and SLSC images. The power Doppler images with adaptive demodulation and SVD filtering show structures unseen in the B-mode and SLSC images, such as clear vessels surrounding the tumor, as shown in Fig. 2a for subject 10. Although the power in the tumor for subject 9 (Fig. 2b) is not necessarily lower than the power in the surrounding liver tissue after TACE in the image with adaptive demodulation and SVD filtering, as is perhaps the case in Fig. 2a,c for subjects 10 and 5, respectively, it is likely that a partial response to treatment was achieved based on the comparison between the post-TACE the pre-TACE images. Additionally, for subject 5, although power in the tumor is faint before TACE with adaptive demodulation and SVD filtering, power is clearly lower in the tumor after TACE compared to surrounding liver tissue when using advanced methods, as shown in Fig. 2c. These observations are not apparent when using conventional methods.

**Figure 3:**
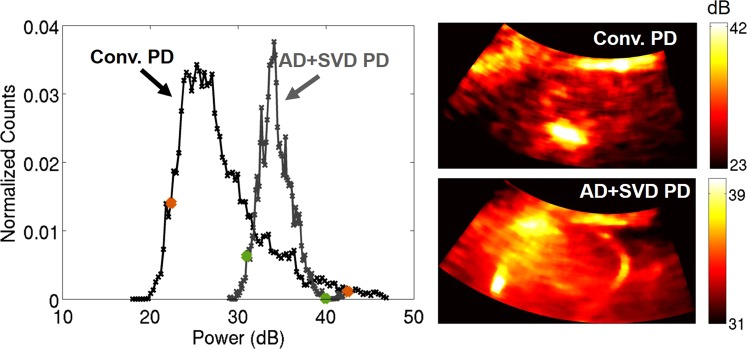
Example dynamic range evaluation for subject 9 after TACE. Histograms are shown on the left for power Doppler (PD) images made with conventional methods (black) (i.e., 16-sample ensemble and IIR filtering) and with adaptive demodulation (AD) and SVD filtering (gray). Histograms were made after log compression. The orange and green dots indicate the dynamic ranges adaptively chosen (middle 70% of the full dynamic range) for the conventional and AD + SVD cases, respectively. Corresponding power Doppler (PD) images are shown on the right on dB scales. PD images were log compressed and cropped to the adaptively selected dynamic ranges.

The dynamic ranges of the displayed power Doppler images are different because of the adaptive scaling approach described in the methods section. However, the dynamic ranges are also inherently different between separate acquisitions (i.e., before and after TACE and between subjects) as well as for the same acquisition when different filters and processing techniques are used. For separate acquisitions, because power Doppler is not globally quantitative, power in one acquisition is not necessarily relevant or correlated to power in another acquisition. For the same acquisition with different processing applied, if the advanced methods are used and are better able to suppress signal from tissue and noise, the dynamic range will be smaller because it will only include the blood signal. If tissue and noise are still present, as can be the case with conventional methods, the dynamic range will be larger because it will include the signal energy from each component (tissue, blood, and noise). Figure 3 demonstrates the potential variability in dynamic ranges and shows histograms and corresponding power Doppler images after TACE for subject 9. Histograms were made of the power Doppler images after log compression. The histograms show how the dynamic range is much larger when conventional methods are used, suggesting that tissue and noise are present. The adaptively chosen dynamic ranges intend to include the majority of each signal, as seen by the orange and green dots on the histograms in Fig. 3 which indicate the adaptive dynamic ranges for the conventional and advanced methods, respectively.

**Figure 4:**
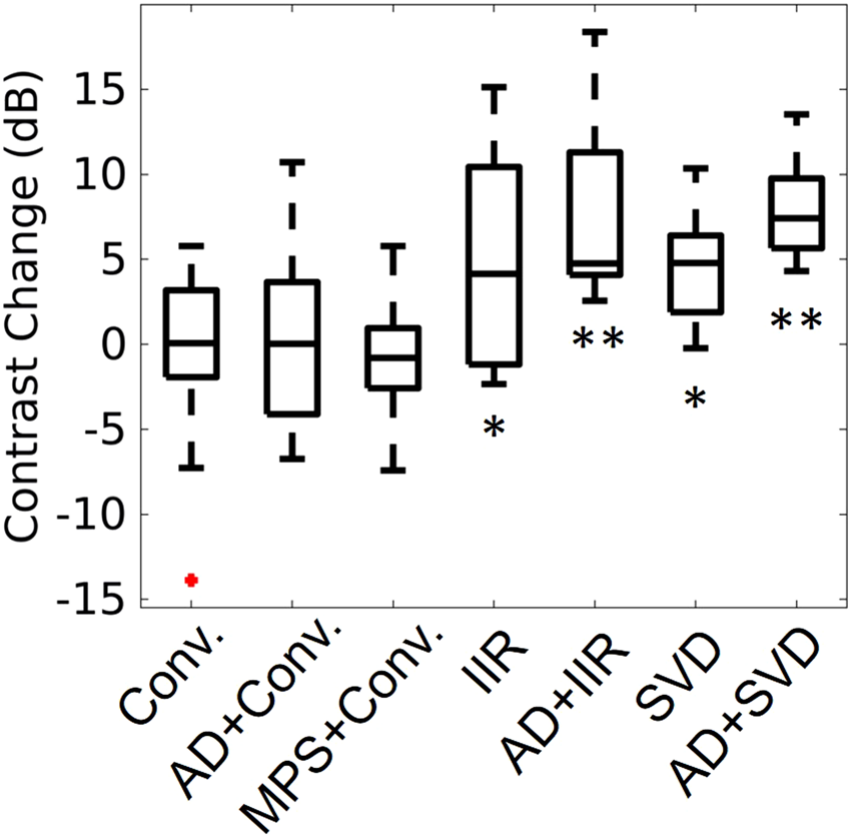
Change in tumor-to-background contrast for each processing technique: Conventional (Conv.), adaptive demodulation (AD) + Conv., mean phase shift (MPS) + Conv., IIR, AD + IIR, SVD, AD + SVD. The median value for each method is the central mark in each box. The 25th and 75th percentiles are the bottom and top edges of each box, respectively. The bars extending from each box indicate the minimums and maximums, and outliers are marked in red. Statistically significant differences from the conventional method are indicated with *(*p* < 0.05) and **(*p* < 0.01).

Quantitatively, changes in contrast were highest when using advanced methods. Figure 4 shows a box plot of the change in contrast for each processing technique. Except for the conventional method which used a 27 ms (16 samples) ensemble size, a 0.5 s (300 samples) ensemble size was used for all other methods. The conventional method by itself, with adaptive demodulation, and with the mean phase shift resulted in the smallest changes in median contrast of 0.06 dB, 0.03 dB, and −0.80 dB respectively. Change in contrast is highest when using adaptive demodulation with SVD filtering which resulted in a median change in contrast of 7.42 dB. Adaptive demodulation with IIR filtering resulted in a notable median change in contrast of 4.76 dB and also resulted in the highest 75th percentile value. Both of these cases also resulted in statistically significant differences from the conventional method with p-values less than 0.01. All post-hoc statistical power values and t-test p-values are displayed in Table 1. All data used for the post-hoc power and t-test were normally distributed as indicated by the Komolgorov-Smirnov test.

**Table 1:** Post-hoc statistical power estimates and p-values for comparisons between each processing method and the conventional method.

Method:	AD + Conv.	MPS + Conv.	IIR	AD + IIR	SVD	AD + SVD
statistical power (%)	12.6	4.1	64.6	94.1	81.9	99.1
p-value	0.434	0.830	0.042	0.005	0.017	0.001

Adaptive demodulation and mean phase shift are abbreviated as AD and MPS, respectively.

**Figure 5:**
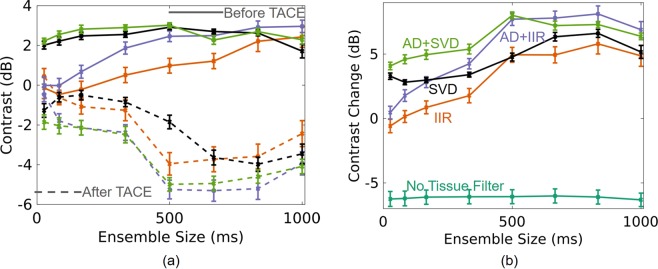
Average contrast (left) and change in contrast (right) for varying ensemble sizes and for each processing method: IIR (orange), adaptive demodulation (AD) + IIR (purple), SVD (black), and AD + SVD (green). On the left, contrast values before and after TACE are shown as the solid and dashed curves, respectively. On the right, change in contrast for when no tissue filtering is used is shown in teal for reference. The no tissue filter values are negative likely because the lipiodol used during TACE is hyperechoic and becomes structure in the tumor.

Compared to standard IIR filtering alone, advanced methods were more robust to varying ensemble sizes. Figure 5a,b show average contrast and average change in contrast, respectively, for each processing method and for ensemble sizes between 27 ms and 1 s (16 and 600 samples). Before TACE, adaptive demodulation with SVD filtering and SVD by itself produce the highest average contrast for ensemble sizes below 600 ms (360 samples), as shown in Fig. 5a. Adaptive demodulation with conventional IIR filtering produces the highest average contrast for the largest ensemble sizes. After TACE, all methods produce lower average contrast when using ensemble sizes above 0.5 s (300 samples), and adaptive demodulation with IIR filtering produces the lowest average contrast overall at a 667 ms (400 sample) ensemble size. These trends are supported in Fig. 5b which shows that adaptive demodulation with SVD filtering produces the highest average change in contrast for smaller ensemble sizes, while adaptive demodulation with IIR filtering produces the largest average change overall when using an 833 ms (500 samples) ensemble size. When no tissue filtering is used, the average change in contrast is negative, indicating that there is increased power in the tumor after TACE. This is likely because of lipiodol in the tumors which shows up as bright B-mode structure and would therefore translate to heightened power in an image without adequate tissue filtering. Statistically, the linear model used to fit the data in Fig. 5b (excluding the no tissue filter data) resulted in an F-statistic corresponding to a p-value less than 0.01, indicating that the model fit the data significantly better than a constant intercept value. Additionally, the model predictors resulted in coefficient values of 12.1, 17.4, 32.7, and 4.56 for SVD (compared to IIR filtering), adaptive demodulation, ensemble size, and the intercept, respectively. These coefficient values suggest that ensemble size is the most important factor in manifesting differences in the change in contrast values from conventional IIR filtering. These values also indicate that adaptive demodulation and SVD filtering strongly influence improvements in changes in contrast values, with adaptive demodulation being more important than SVD.

**Figure 6:**
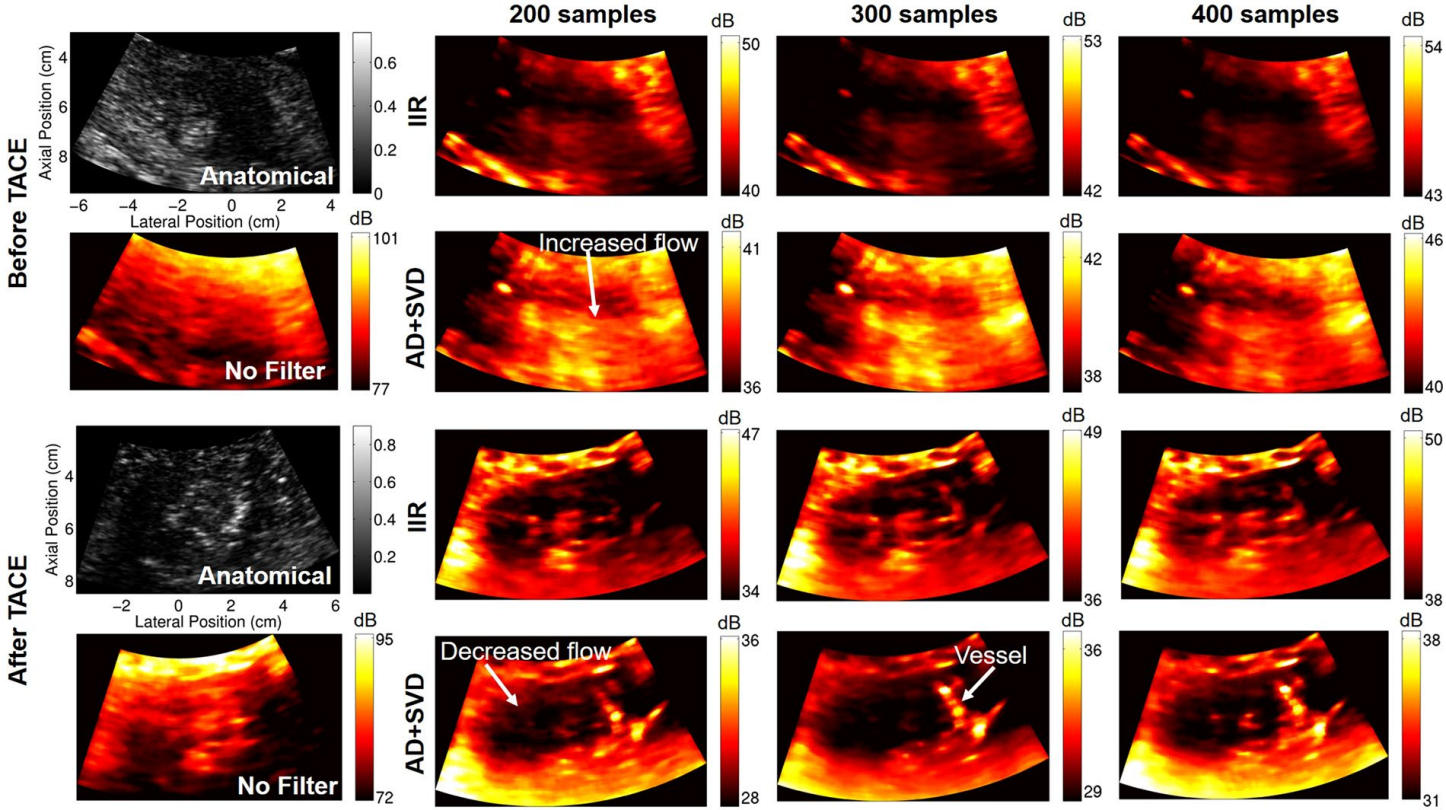
Power Doppler images of subject 1 before TACE (top) and after TACE (bottom). Power Doppler images are made using no tissue filtering, IIR filtering, and with adaptive demodulation (AD) and SVD filtering for ensemble sizes of 200, 300, and 400 samples (333 ms, 500 ms, and 667 ms). Focused SLSC images are included for anatomical reference.

To support the trends in Fig. 5 qualitatively, Fig. 6 shows example power Doppler images of subject 1 using conventional IIR filtering and using adaptive demodulation with SVD filtering for ensemble sizes of 333 ms, 500 ms, and 667 ms (200, 300 and 400 samples). Before TACE, the power Doppler images made with conventional IIR filtering show minimal signal inside the tumor, while the images made with adaptive demodulation and SVD filtering show enhanced power in the tumor compared to when no tissue filtering is used. After TACE, both methods show suppressed power in the tumor, but IIR filtering fails to suppress all of the signal seen in the anatomical SLSC and no filter images. In contrast, the images made with adaptive demodulation and SVD filtering show suppressed signal in the tumor, especially when using the 0.5 s (300 samples) ensemble size. Also a clear vessel is seen lining the tumor in the images made with adaptive demodulation and SVD filtering that is not seen in the images made with IIR filtering, no filtering or the anatomical SLSC images.

## Discussion

The results demonstrate that adaptive demodulation in combination with advancements in beamforming and filtering can adequately detect changes in perfusion after TACE. More specifically, adaptive demodulation with SVD filtering resulted in the largest median changes in contrast between time points, which is indicative of successful occlusion of the tumor arterial supply. These changes were far less apparent with conventional methods.

Because our conventional method uses synthetic transmit aperture beamforming to focus at all locations in the image, it should have better resolution and SNR than a true conventional power Doppler acquisition implemented with single focus transmits. However, because we use plane waves and acquire our data with a research scanner, we will likely not have SNR that is comparable to what is commercially available. Future studies could incorporate a truly conventional power Doppler scan using a commercial scanner to address this, but the improved SNR that comes from using a clinical platform would also improve the advanced methods.

The results are supported by previous work that showed the benefit of using adaptive demodulation and SVD compared to IIR filtering alone in simulations, *in vitro* phantom, and preliminary *in vivo* experiments^37,38^. However, most of those studies tested these techniques at a 7.8 MHz imaging frequency and at depths only down to 2.5 cm^37,38^. Here, we show that these methods can be effective at a clinical imaging frequency of 4.2 MHz and down to 10 cm. Moreover, compared to other studies that have shown the benefit of SVD filtering in fairly stationary and shallow organs like transplanted kidneys^33^, we show here that these methods can be successfully applied in the larger and more mobile liver.

In the same previous work, we showed that adaptive demodulation is particularly useful when using smaller ensemble sizes^37^, which is what we observed in this work as well. To minimize processing time and facilitate real-time applications, smaller ensemble sizes are preferred. In this work, a 0.5 s ensemble size actually produced higher changes in contrast than the full 1 s ensemble for most techniques, which could be due to unoptimized filtering or decorrelation effects due to large physiological or sonographer hand motion. Regardless, in this work, a 0.5 s ensemble size proved to be the smallest and most effective ensemble size for visualizing both qualitative and quantitative changes in blood flow before and after treatment.

In addition to facilitating earlier TACE treatment evaluation, a real-time implementation of the proposed techniques is desirable because we currently rely on anatomical B-mode to select the best field of view of the tumor, which does not necessarily correspond to the best Doppler field of view. Although development of a real-time implementation was not the focus of this work, we believe it is possible with alternative processing techniques and improved hardware. In this work, the full 1 s ensemble size was beamformed in the time-domain on a single CPU and was the most computationally expensive step. However, apart from implementing a more parallelized approach using smaller ensemble sizes, GPU and Fourier-based methods^39–41^ have been shown to be able to facilitate synthetic aperture beamforming in real-time. Additionally, GPU-based autocorrelation methods have been proposed for real-time motion estimation^42^ which could allow for real-time implementations of adaptive demodulation. Finally, real-time SVD implementations through the use of block-wise approaches and multi-core CPUs have also been proposed^43^. Therefore, with reasonably short ensemble sizes, we believe that implementing our proposed method in real-time is possible and will be the focus of future work.

This work intends to showcase initial feasibility of using non-contrast ultrasound as a tool to evaluate TACE. To do this, we made the assumption that treatment was on average successful, which, based on the cited response rate of 62–76%^6,7^, is unlikely. Therefore, the tumor and background ROIs were drawn to avoid potential incomplete response regions in an effort to depict general changes in perfusion. Differentiating between true therapeutic responses (i.e., complete, partial, or incomplete response) was not the intention of this work, but there were potential residual tumor or blood flow regions observed in our small pilot study that will need to be confirmed as real or artifact with gold-standard follow-up imaging. For this study, gold-standard follow-up imaging has not been acquired for all of the patients. Thus, a necessary future study will aim to statistically compare our proposed non-contrast ultrasound technique to CE-MR or CE-CT.

We include preliminary statistics in this work to support the study sample size and to indicate statistically significant differences between each technique and the conventional method. With the proposed combination of adaptive demodulation and SVD, we observed the highest statistical power and significance, suggesting that 11 subjects for an initial pilot study is sufficient.

Non-contrast ultrasound has several advantages over CE-US, CE-MRI, and CE-CT. It is completely noninvasive, more affordable, more accessible, and it is not constrained by time and dose of contrast agents. Therefore, if non-contrast ultrasound has the potential to have similar if not better performance to these other evaluation techniques, it could be an invaluable addition to current treatment evaluation. Additionally, non-contrast ultrasound is not affected by the same treatment-induced imaging artifacts as with CE imaging and could potentially be used immediately after or during the procedure, facilitating re-treatment during the same session. Digital subtraction angiography (DSA) is currently used during the procedure to guide and assess treatment delivery^8^. However, DSA is limited to visualization of the vessel architecture that has been injected with contrast, potentially leaving collateral tumor feeder vessels undetected, which could lead to a partial or incomplete response to treatment^44^. Non-contrast ultrasound blood flow estimation could detect these collateral feeder vessels because it is sensitive to blood flow rather than contrast agents. Therefore, the proposed technique could provide the necessary complimentary monitoring and evaluation of treatment.

## Methods

### Patients and procedure

This study was approved by and performed in accordance with the relevant guidelines and regulations of Vanderbilt’s institutional review board. Ten patients undergoing TACE gave informed written consent to participate. One of the patients received TACE twice (3 months apart) and consented twice, resulting in a total of 11 subject acquisitions. Inclusion criteria consisted of the patient being over 21 years of age with a scheduled TACE procedure and the ability to provide informed consent. All patients received conventional TACE. The ultrasound acquisitions did not alter the treatment protocol or impose any additional risk for which ethical committee authorization would be necessary. Table 2 summarizes patient demographics and tumor information.

**Table 2:** Summary of patient demographics and tumor characteristics for each subject acquisition.

Subject	Age (y)	Sex	BMI $(\tfrac{{\boldsymbol{kg}}}{{{\boldsymbol{m}}}^{{\bf{2}}}})$	Type	Size (cm)	Location
1	56	M	47.4	HCC	4.7	Segment 3
2	52	F	23.0	HCC	2.0	Segment 5/6
3	55	M	24.4	NET	6.3	Segment 6
4*	60	F	27.3	HCC	3.4	Segment 5
5	58	M	25.8	HCC	2.4	Segment 5
6	43	F	25.0	NET	6.0	Segment 6/7
7	64	F	22.4	NET	4.1	Segment 4B
8*	60	F	27.3	HCC	4.5	Segment 5
9	60	M	32.5	HCC	3.4	Segment 5/6
10	73	M	21.7	NET	6.0	Segment 6
11	63	M	24.4	HCC	5.3	Segment 6

Tumor types consisted of neuroendocrine tumors (NET) and hepatocellular carcinomas (HCC). Tumor size is indicated as the largest measured diameter.

*Same patient.

### Imaging

**Figure 7:**
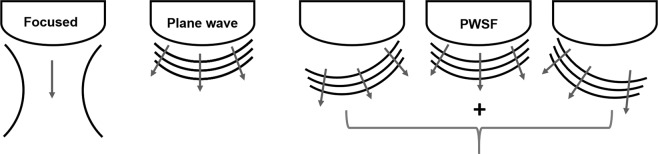
Cartoon depiction of the different transmit sequences used in this study. Focused scans acquire a single lateral location of an image at a time and are focused at a single depth. Plane wave scans sacrifice transmit focusing and involve transmitting from all elements at once to insonify the entire field of view. Plane wave synthetic focusing (PWSF) involves transmitting multiple angled plane waves and then summing them to gain transmit focusing throughout the image (i.e., at all depths and lateral locations).

A Verasonics C5-2 probe with a 4.2 MHz center frequency was used to acquire a 6 cm focused anatomical scan followed continuously by 2 s of an angled plane wave Doppler sequence. For both the focused anatomical and unfocused Doppler sequences, the channel data were acquired and saved immediately before and after TACE for each patient. Compared to a focused scan which acquires a single line at a time, plane wave imaging insonifies the entire field of view at once, as depicted in Fig. 7. In the case of a curvilinear array, the wave is more spherical than planar, but we use the term “plane wave” to be consistent with the literature. With respect to Doppler techniques, plane wave imaging overcomes the long-standing trade-off between frame rate and ensemble length with focused Doppler^29^ and is discussed in more detail in the following section. The plane wave sequence used 9 angles between −8° and 8° at a PRF of 5.4 kHz. Both sequences imaged down to 10 cm. Imaging was performed by the interventional radiologist performing the procedure. When possible, patients were asked to hold their breath during each scan.

### Post-processing

#### Beamforming

For the focused anatomical sequence, conventional delay-and-sum beamforming was used to generate anatomical B-mode images. SLSC beamforming was also implemented on the focused data for additional and sometimes improved anatomical referencing^45^. A maximum aperture lag of 10 elements and an axial kernel size of 1.5*λ* (*λ* = center frequency wavelength) were used for SLSC beamforming. For both delay-and-sum and SLSC, Hann apodization and aperture growth to achieve an F/# of 2 were implemented during receive beamforming.

For the unfocused (i.e., plane wave) Doppler sequence, plane wave synthetic focusing was implemented as in Montaldo *et al*.^30^, resulting in a frame rate of 600 Hz after beamforming. Plane wave synthetic focusing, as depicted in Fig. 7, combines beamformed consecutive angled plane wave data to achieve synthetic transmit focusing throughout the image^30^. This differs from true focused imaging which only focuses at a single location on transmit. Hann apodization and aperture growth to achieve an F/# of 2 were implemented during receive beamforming. Beamformed data were up-sampled and band-pass filtered. Up-sampling was performed to achieve a sampling frequency of 50 MHz.

For tissue filtering and power Doppler estimation (described in following subsections), a 1 s (600 sample) ensemble (either the first or second half of the full 2 s ensemble) was qualitatively chosen based on M-mode motion for each data set. The first 0.5 s of each 1 s ensemble was used for initial qualitative and quantitative comparisons between patients and filtering techniques. An ensemble size evaluation was also performed for which ensembles between 83 ms and 1 s (50 and 600 samples) were compared. These long ensemble sizes are made possible by unfocused plane wave sequences. Conventional focused Doppler sequences acquire Doppler ensembles for a single line at a time, resulting in a trade-off between frame rate and ensemble length. Therefore, conventional methods typically use 16 samples or less for a Doppler ensemble^29^. Because we did not acquire a traditional focused Doppler scan, to mimic a conventional scan, a 16-sample ensemble was also evaluated and will be referred to as the “conventional” method. However, it is worth noting that because we are using plane wave synthetic focusing to synthetically focus at all locations on transmit rather than a single location as is done with true conventional techniques, our “conventional” method should still be better than traditional power Doppler imaging as currently implemented on most commercial scanners. It is also worth noting that the conventional data and the longer ensemble IIR data are equivalent apart from the ensemble size.

#### Adaptive demodulation

Adaptive demodulation was applied as in Tierney *et al*.^32^ to the beamformed plane wave data. To account for axial tissue motion, phase demodulation was implemented using a 10*λ* (*λ* = center frequency wavelength) axial kernel size and lag of 1 slow-time sample (1.7 ms) to compute relative and total tissue displacements through slow-time, respectively. These parameters were chosen based on previous work^37^. Amplitude demodulation did not provide any additional benefit and was therefore not applied.

A mean phase shift (i.e., frequency down-mixing) approach was also implemented on the conventional sequence for which there may be a substantial mean tissue frequency other than zero due to the short ensemble length. This was implemented the same way as was done in Bjaerum *et al*.^24^.

#### Tissue filtering

Each data set was cropped to a smaller field of view prior to tissue filtering. A conventional IIR filter as well as an adaptive SVD filter were applied to each data set. For the IIR filter, a 30 Hz (5.5 mm/s) high-pass 6th order type 1 Chebyshev filter was used. A symmetric initialization was performed for which 20 mirrored samples (16 samples for the conventional case) were added to each slow-time signal before filtering and removed after. The IIR filter used was chosen based on previous studies^23^ as well as internal comparison to other IIR and FIR filters. For the SVD filter, an adaptive 2D spatio-temporal approach was used as in Song *et al*.^36^. The same thresholds were used for all data sets for adaptively determining tissue and noise cutoffs. Two tissue cutoffs were computed and the maximum or minimum were used based on which produced the best qualitative and quantitative result for the 0.5 s (300 sample) ensemble for each data set.

#### Image evaluation

Power Doppler was computed on each data set using

1 $$PD(x,z)=\mathop{\sum }\limits_{t=1}^{T}\,|s(x,z,t){|}^{2}$$

where $s(x,z,t)$ is the filtered analytic signal, *x*, *z*, *t* are the spatial, axial, and temporal dimensions, and *T* is the total number of slow-time samples (i.e, ensemble size). A 2 mm by 2 mm spatial median filter was applied to each power Doppler image. Aperture growth effects were accounted for by dividing each power Doppler value at a given depth by the number of aperture positions used for aperture growth at that depth.

To quantify differences between before and after TACE time points as well as differences between processing techniques, we use a tumor-to-background contrast metric as follows,

2 $$C=10lo{g}_{10}\frac{\frac{1}{N}\,{\sum }_{i=1}^{N}\,P{D}_{tumor}(i)}{\frac{1}{M}\,{\sum }_{i=1}^{M}\,P{D}_{bkgd}(i)}$$

where *N* and *M* are the total number of pixels in the tumor and background, respectively, and *PD*_*tumor*_ and *PD*_*bkgd*_ are the power Doppler values in the tumor and background, respectively. Contrast provides a relative measurement of spatial changes in power between the tumor and surrounding liver tissue. To measure temporal changes, change in contrast was computed as the difference between time points (before minus after) for each patient. Power amplitude can vary between acquisitions due to different noise characteristics and fields of view, which can make comparisons between before and after TACE challenging. However, because contrast is a relative metric to each individual image, comparing temporal changes in contrast is feasible. Furthermore, these quantitative metrics are independent of how the images are scaled for qualitative display. Example tumor and background masks are shown in Fig. 1. Masks were manually drawn for each data set using the power Doppler images in addition to the anatomical B-mode and SLSC images.

Assuming successful treatment, we expect decreased power (i.e., perfusion) in the tumor after TACE. We also expect potentially elevated power in the tumor before TACE due to tumor hypervascularity^5,8,11^. Based on this hypothesis, we expect contrast to be positive before TACE, which would suggest increased blood flow in the tumor relative to the background tissue. Conversely, we expect contrast to be negative after TACE if blood flow in the tumor has been effectively occluded. Therefore, we expect the change in tumor contrast to be positive and greater than the before TACE contrast value, which would mean that the power in the tumor decreased after TACE relative to the power in the tumor before TACE.

Displayed power Doppler images were made by log compressing (1) ($I=10{lo}{{g}}_{10}PD(x,z)$). Dynamic ranges were chosen to include the middle 70% of the full dynamic range (i.e., the top and bottom 15% were set to the maximum and minimum values, respectively).

#### Statistics

To test for significant differences between the filtering techniques and the conventional methods, p-values were computed using a two-sample paired t-test on the change in contrast values. Significance was determined based on *α* = 0.05. Additionally, as a supplemental indication of effect size, post-hoc statistical power was computed on the change in contrast values for comparisons between each filtering technique and the conventional method. The cutoff used for the power calculation was *α*/2 because we conducted a two-sided test. For the purposes of this statistical power calculation we assumed that both the null and alternative hypotheses were normally distributed even though the actual statistical test for significance used the t-distribution. This assumption will result in estimates of statistical power that are slightly higher than the true power. A Komolgorov-Smirnov test was used to test for normality. Furthermore, to determine relative contributions to differences between techniques, a linear regression model was fit to the change in contrast values for which ensemble size, adaptive demodulation, and SVD were used as predictors. To facilitate direct comparisons, each predictor was centered about the mean and normalized by overall power (i.e., square root of the mean of the squared values). An F-test was used to assess the model fit.

#### Processing details

All post-processing was done in MATLAB (The MathWorks, Inc., Natick, MA). All Doppler sequence beamforming and adaptive demodulation were performed using Vanderbilt’s Advanced Computing Center for Research and Education (ACCRE). Parallel CPUs were used on ACCRE to beamform 1 s (5400 plane wave acquisitions) of channel data per CPU and to compute slow-time relative displacements for adaptive demodulation for a single beamformed line per CPU. Beamforming and adaptive demodulation per 1 s ensemble took approximately 30 minutes. All tissue filtering and power Doppler estimation were performed on local CPUs and took approximately 1 minute for a 300-sample ensemble with SVD filtering. IIR filtering and smaller ensemble sizes took less time.

## Conclusion

Curative treatments are rarely available for hepatic malignancies, and palliative management of these diseases are often necessary with TACE treatment. However, TACE is variably effective and evaluation is limited by late follow-up imaging. We propose non-contrast perfusion ultrasound imaging as a solution for immediate, and potentially intra-procedural, treatment evaluation. We show preliminary feasibility of our proposed technique in a small pilot study. Our results indicate that combining slow flow techniques could potentially make non-contrast perfusion imaging a viable tool for evaluating TACE.
